# Infarct-to-spleen volume ratio as a novel volumetric predictor of splenectomy in splenic infarction

**DOI:** 10.1186/s12893-025-03457-9

**Published:** 2026-01-10

**Authors:** Abdullah Gunes, Nuray Colapkulu-Akgul, Ibrahim Unlu, Mehmet Furkan Avcı, Almotasem Shatat, Ahmet Yalnız, Saffet Cınar, Zafer Utkan

**Affiliations:** 1 Department of General Surgery, Kocaeli City Hospital, Kocaeli Sehir Hastanesi, Izmit, 41060 Kocaeli Türkiye; 2 Department of General Surgery, Gebze Fatih State Hospital, Kocaeli Sehir Hastanesi, Izmit, 41060 Kocaeli Türkiye; 3 Department of General Surgery, Kocaeli University Medical Faculty, Kocaeli, Türkiye; 4 Department of Radiology, Kocaeli University Medical Faculty, Kocaeli, Türkiye; 5 Department of General Surgery, Derince Training and Research Hospital, Kocaeli, Türkiye

**Keywords:** Splenic infarction, Splenectomy, Infarct-to-spleen volume ratio, Volumetric analysis, Risk stratification, Computed tomography

## Abstract

**Background:**

Splenic infarction is a rare and often underrecognized condition with diverse etiologies and variable clinical presentations. While most cases are managed conservatively, identifying patients who may require surgical intervention remains a clinical challenge. This study aimed to evaluate the infarct-to-spleen volume ratio (ISR) as a radiologic predictor for splenectomy in patients with splenic infarction and to propose a risk stratification model incorporating ISR and fever.

**Methods:**

In this retrospective, two-center cross-sectional study, 236 patients diagnosed with splenic infarction between January 2015 and January 2023 were included. Volumetric analysis was performed using contrast-enhanced CT to calculate ISR. Clinical, laboratory, and radiologic features were compared between surgical (*n* = 13) and non-surgical (*n* = 223) groups. ROC curve and logistic regression analyses were used to evaluate predictive parameters for splenectomy. A risk score based on ISR and fever status was developed to stratify patients into risk categories.

**Results:**

The splenectomy rate was 5.6%. Patients who underwent splenectomy had significantly higher ISR (median 28.2 vs. 9.17, *p* = 0.02). ROC analysis identified ISR as a strong predictor for surgery (AUC = 0.69, 95% CI: 0.49–0.86), with a specificity of 77.9% and NPV of 97.2%. Multivariate logistic regression confirmed ISR (OR: 1.03, *p* = 0.002) and fever (OR: 2.69, *p* = 0.005) as independent predictors. A risk score (range 0–4) based on these variables stratified patients into low-, intermediate-, and high-risk groups with splenectomy rates of 1.5%, 6.3%, and 33.3%, respectively.

**Conclusion:**

ISR is a novel, objective predictor of the need for splenectomy in patients with splenic infarction. Incorporating ISR and fever into a simple risk score may aid clinical decision-making and help identify candidates for early conservative management or outpatient follow-up.

## Introduction

Splenic infarction is a relatively uncommon and often underdiagnosed clinical entity resulting from compromised blood flow to the splenic parenchyma. The condition is most commonly associated with thromboembolic or hypercoagulable states such as atrial fibrillation, hematologic malignancies, and infectious or autoimmune disorders [1–5]. Clinical presentation varies widely, ranging from asymptomatic incidental findings to acute left upper quadrant pain and systemic inflammatory responses [4, 6–9]. Despite increasing recognition through advanced imaging modalities, particularly contrast-enhanced computed tomography (CT), the diagnosis is frequently delayed due to its nonspecific and variable clinical manifestations [9, 10]. Although the majority of splenic infarctions are managed conservatively, surgical intervention, particularly splenectomy, may be required in selected cases, typically in the presence of complications such as rupture, abscess, or persistent symptoms [5, 6, 9, 10]. However, reliable clinical or radiologic predictors for identifying patients at risk for surgical intervention remain poorly defined. Previous studies have primarily focused on clinical presentation, laboratory abnormalities, and underlying etiologies, while few have quantitatively evaluated infarct burden as a predictive factor for surgery. In this context, volumetric analysis offers an objective approach to measuring the extent of parenchymal damage. While the infarct-to-spleen volume ratio (ISR) has been studied in the setting of partial splenic embolization to guide therapeutic efficacy and minimize complications, its predictive role in spontaneous splenic infarction has not been previously investigated. The present study aims to evaluate the association between ISR and the need for splenectomy in patients with splenic infarction, and to identify ISR as a potential quantitative radiologic predictor for surgical decision-making.

## Materials and methods

### Study design

**Fig. 1:**
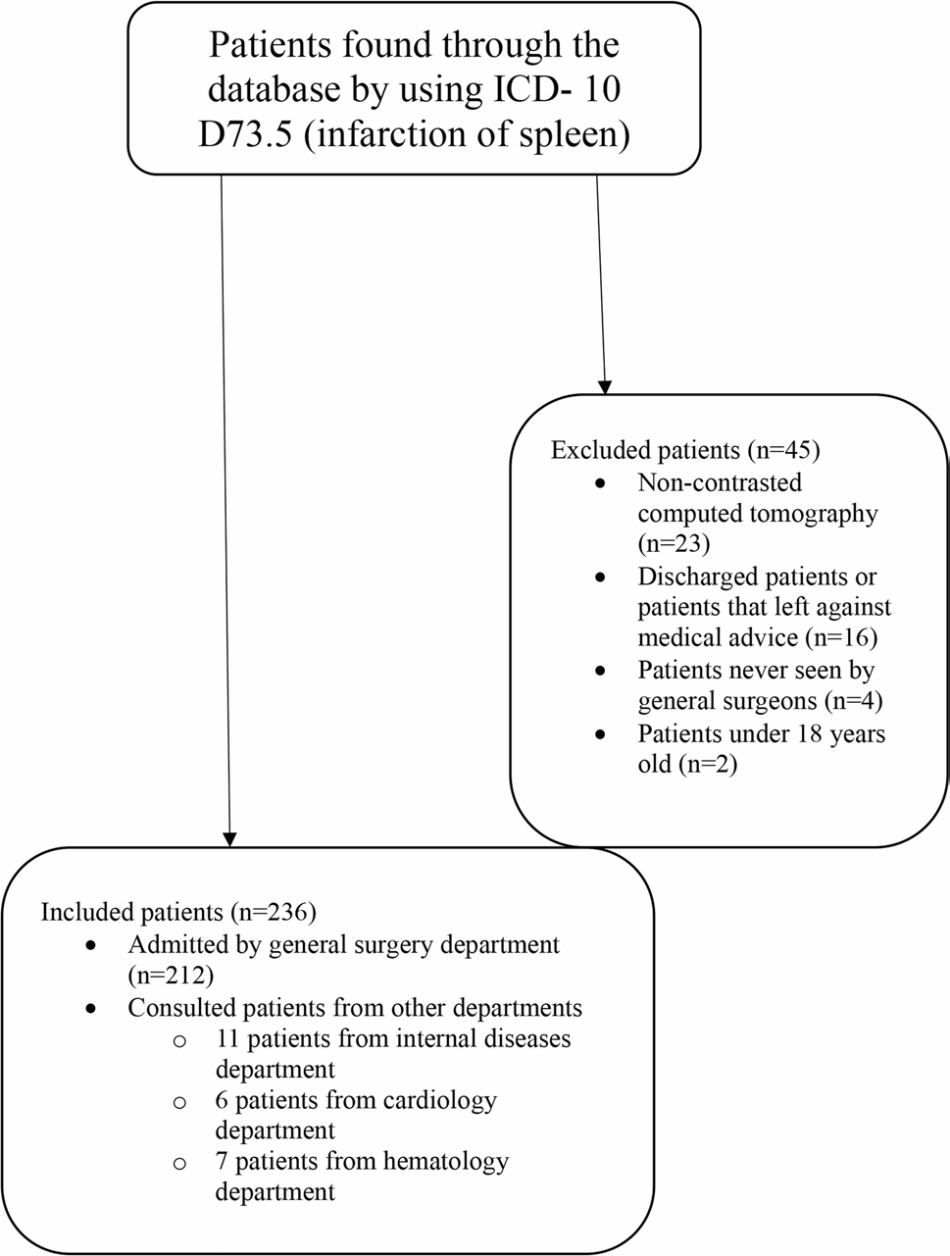
Patients flow

This was a retrospective, two-center, cross-sectional study including patients diagnosed with splenic infarction between January 2015 and January 2023 at Departments of General Surgery, Kocaeli Derince Research and Training Hospital and Kocaeli University Medical Faculty, Turkiye, were included. Patients were identified through a database search using the International Classification of Diseases [ICD- 10 D73.5 (infarction of spleen)]. Clinical, laboratory, and radiologic data were retrospectively collected from hospital database. Inclusion criteria were: being older than 18-year-old, presence of intravenous contrasted computer tomography, being admitted to general surgery department or consulted patients from other departments. Exclusion criteria were: CT without contrast, patients that were not admitted to hospital or followed up by general surgery department (Fig. 1).

Patients were categorized into two groups for comparative analyses based on whether they underwent surgery: those who underwent splenectomy (surgical group) and those who did not (non-surgical group). All patients received antibiotics and anticoagulant therapy with low-molecular-weight heparin. In cases where abdominal pain was present, non-steroidal anti-inflammatory drugs or narcotic were also administered. Age, gender, complete blood count values and biochemical parameters and possible etiological comorbidities are recorded. Neutrophil-to-lymphocyte ratio (NLR), platelet-to-lymphocyte ratio (PLR) and white blood cell (WBC)-to-lactate dehydrogenase (LDH) ratio are also calculated. Body temperature was stratified into three categories: afebrile, defined as < 37.5 °C; subfebrile, defined as 37.5–38.0 °C; and febrile, defined as > 38.0 °C.

**Fig. 2:**
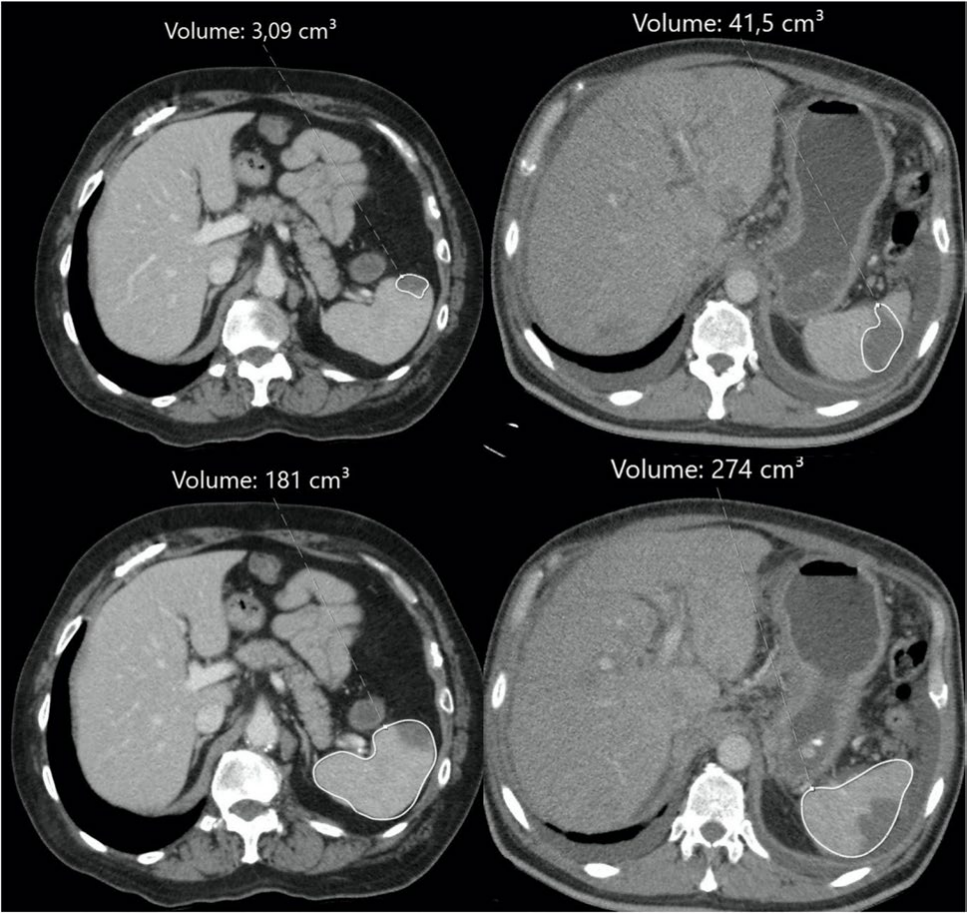
Axial contrast-enhanced abdominal CT images demonstrating splenic infarction with volumetric analysis

Volumetric measurements of the spleen and splenic infarcts were performed using a Sectra PACS (Sectra AB, Linköping, Sweden). All measurements were based on contrast-enhanced abdominal CT scans acquired in the portal venous phase using a 128-slice multidetector CT scanner (Toshiba/Canon Medical Systems). On each axial slice, the spleen was manually contoured to allow for volumetric reconstruction (Fig. 2). The total splenic volume was automatically calculated by the software through summation of the areas across all slices. The infarcted areas were also manually segmented using the same method, and infarct volumes were calculated separately. All segmentations were independently performed by a radiology resident and a board-certified radiologist with over 10 years of experience. Discrepancies were resolved by consensus.

### Model Building and validation

Variables with a univariate association at *p* < 0.10 were evaluated for inclusion. A multivariable logistic regression model was then constructed, and independent predictors were retained in the final model if they remained statistically significant at *p* < 0.05. Model performance was evaluated in terms of both discrimination and calibration. Discrimination was quantified by calculating the area under the receiver operating characteristic curve (AUC) with 95% confidence intervals, while calibration was assessed using the Hosmer–Lemeshow goodness-of-fit test with 10 groups (*p* > 0.05 indicating acceptable fit). The overall accuracy of the model was further described using the Brier score.

### Outcomes

Primary outcome of this study is to evaluate whether the ISR is an independent predictor for splenectomy in patients with splenic infarction. Secondary outcome is to develop and internally validate a risk stratification model based on ISR and fever status for predicting surgical need.

### Statistical analyses

Continuous variables were expressed as median and interquartile range (IQR), while categorical variables were presented as frequency and percentage. The Mann–Whitney U test was used for comparisons of non-normally distributed continuous variables between surgical and non-surgical groups. The Chi-square test was applied to assess associations between categorical variables. Subgroup comparisons were evaluated using the Kruskal–Wallis test followed by post hoc pairwise comparisons. Receiver operating characteristic (ROC) curve analysis was conducted to evaluate the predictive performance of hematologic and clinical parameters, including C-reactive protein, WBC/LDH, NLR, PLR, and ISR, for surgical intervention. The optimal cut-off values for each parameter were determined using the Youden Index, which identifies the threshold that maximizes both sensitivity and specificity. To evaluate diagnostic performance, the area under the ROC curve (AUC) was calculated, and 95% confidence intervals (CIs) were estimated using DeLong’s method. Diagnostic metrics — including sensitivity, specificity, positive predictive value (PPV), negative predictive value (NPV), and overall accuracy — were derived from standard 2 × 2 contingency tables. Confidence intervals for these proportions were computed using the Wilson score method, which provides robust estimates for binomial outcomes. Univariate and multivariate logistic regression analyses were performed to identify independent predictors of surgery. Variables with *p* < 0.01 in univariate analysis were included in multivariate models. Statistical analyses were performed using Python version 3.11 (Python Software Foundation, USA) and R version 4.2.1 (https://www.r-project.org/)., where appropriate. A two-sided *p*-value < 0.05 was considered statistically significant.

## Results

### Clinical and etiological features

**Fig. 3:**
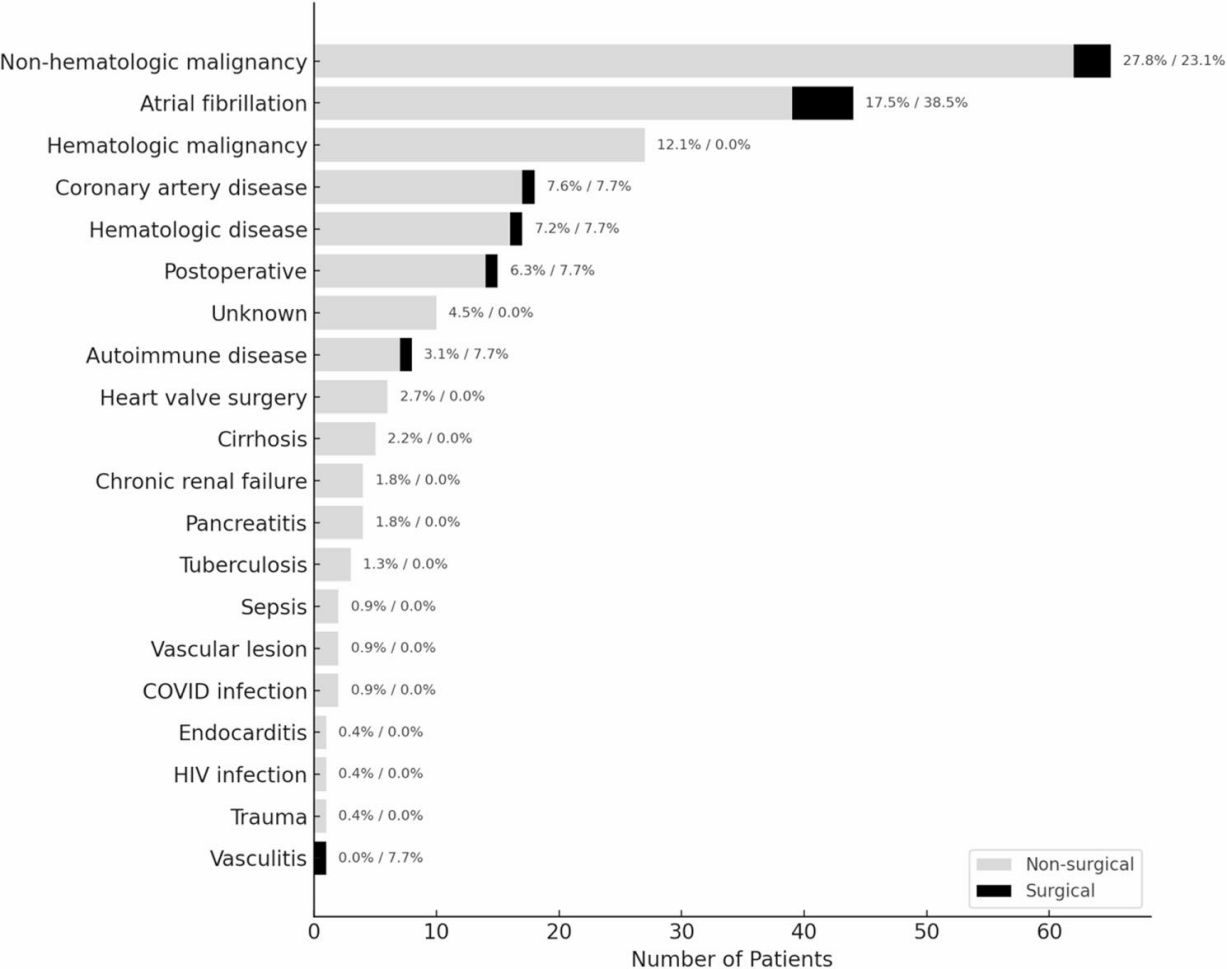
Distribution of underlying etiologies in patients with and without splenectomy

A total of 236 patients diagnosed with splenic infarction were included in the study. Of these, 13 patients (5.6%) underwent splenectomy. The median age of the study population was 64 years [20–96] for non-surgical group and 58 [18–90] for surgical group, and 55.5% (*n* = 131) were male. The most common predisposing condition was non-hematologic malignancy (27.9%), followed by atrial fibrillation (18.4%) and hematologic malignancy (13.1%) (Fig. 3). While the majority of patients without surgery reported diffuse abdominal pain (57.3%), those who underwent surgery most commonly presented with left upper quadrant pain (69.2%) (*p* = 0.612). Forty-four patients (19.4%) without abdominal pain were consulted due to incidental splenic infarction, and leading causes were non-hematologic malignancies (*n* = 13, 29.5%) and cardioembolic events (*n* = 9, 20%). Additionally, patients who underwent surgery more frequently presented with febrile (38.4%) or subfebrile (23.2%) states compared to those without surgery (*p* = 0.002) (Table 1).

**Table 1:** Comparison of demographic and clinical features between surgical and non-surgical groups

	Non-surgical	Surgical	*P* values
	*n* * = 223 (94.4%)*	*n* * = 13 (5.6%)*	
Age, *median (min-max)*	64.0 (20.0–96.0)	58.0 (18.0–90.0)	0.329
Sex, *(m/f)*	123 (%55.2) / 100 (%44.8)	8 (%61.5) / 5 (%38.5)	0.070
Abdominal pain, *n (%)*			0.612
Absent	44 (19.7)	0 (0.0)	
Left upper quadrant	51 (23)	9 (69.2)	
Diffuse	128 (57.3)	4 (30.8)	
Fever, *n (%)*			**0.002**
Absent	167 (74.8)	5 (38.4)	
Subfebrile	34 (15.2)	3 (23.2)	
Febrile	21 (10.0)	5 (38.4)	
Follow up (days), *median (IQR)*	2 (3.5)	18 (15)	**0.004**

### Infarct-to-spleen volume ratios and radiological features

**Fig. 4:**
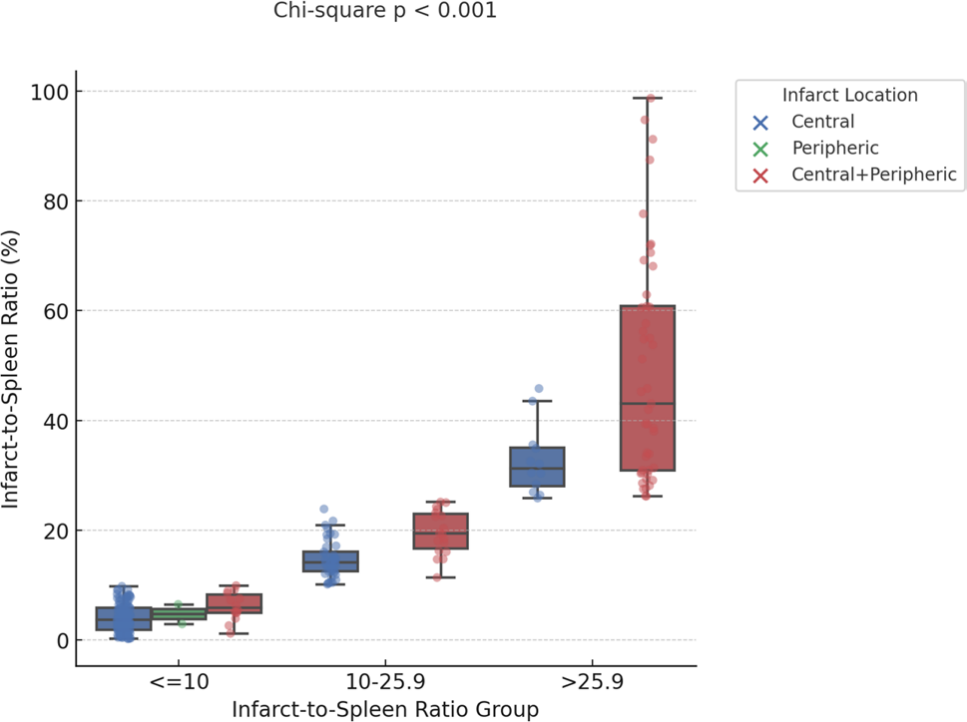
Distribution of infarct location across infarct-to-spleen volume ratio groups

Table 2 reveals the comparison radiological metrics between two groups. The median splenic volume was higher in the surgical group compared to the non-surgical group (442.0 [IQR: 270.0–853.0] vs. 317.0 [IQR: 203.0–611.5]) (*p* = 0.167). In contrast, both infarct volume and ISR were significantly greater in patients who underwent surgery. The median infarct volume was 152.0 (IQR: 53.0–189.0) in the surgical group versus 36.0 (IQR: 10.2–80.5) in the non-surgical group (*p* = 0.01). Similarly, the ISR was significantly higher in the surgical group (28.2 [IQR: 10.51–45.89]) than in the non-surgical group (9.17 [IQR: 4.03–22.66]; *p* = 0.02). To determine a clinically relevant lower threshold for risk analysis, this categorization of ISR (< 10, 10–25.9, > 25.9) was included as a variable in the logistic regression model. Regarding infarct location, a central pattern was more common in the non-surgical group (68.1%) compared to the surgical group (38.5%), while combined central and peripheral infarcts were more frequently observed in patients who underwent surgery (61.5% vs. 30.9%). A significant association was found between ISR groups and infarct location, with central infarction being more frequent in the ≤ 10 group and combined central and peripheric involvement predominating in the > 25.9 group (Chi-square *p* < 0.001) (Fig. 4).

**Table 2:** Comparison of radiological volumetric metrics between surgical and non-surgical groups

	Non-surgical	Surgical	*P* values
	*n* * = 223 (94.4%)*	*n* * = 13 (5.6%)*	
Spleen volume (cm^3^), *median (min-max)*	317.0 (18.0-5857.0)	442.0 (161.0-1180.0)	0.167
Infarct volume (cm^3^), *median (min-max)*	36.0 (10.2–80.5)	152.0 (53.0–189.0)	**0.01**
Infarct-to-spleen volume ratio (cm^3^), *median (min-max)*	9.17 (0.2–94.8)	28.2 (0.5–98.8)	**0.02**
Infarct-to-spleen volume ratio (cm^3^), *n (%)*			**0.00**
≤10	117 (52.4)	3 (23.0)	
10–25.9	57 (25.5)	2 (15.5)	
>25.9	49 (22.1)	8 (61.5)	
Infarct location, *n (%)*			0.070
Central	152 (68.1)	5 (38.5)	
Peripheric	2 (1.0)	0 (0.0)	
Central + peripheric	69 (30.9)	8 (61.5)	

### ROC and logistic regression analyses

**Fig. 5:**
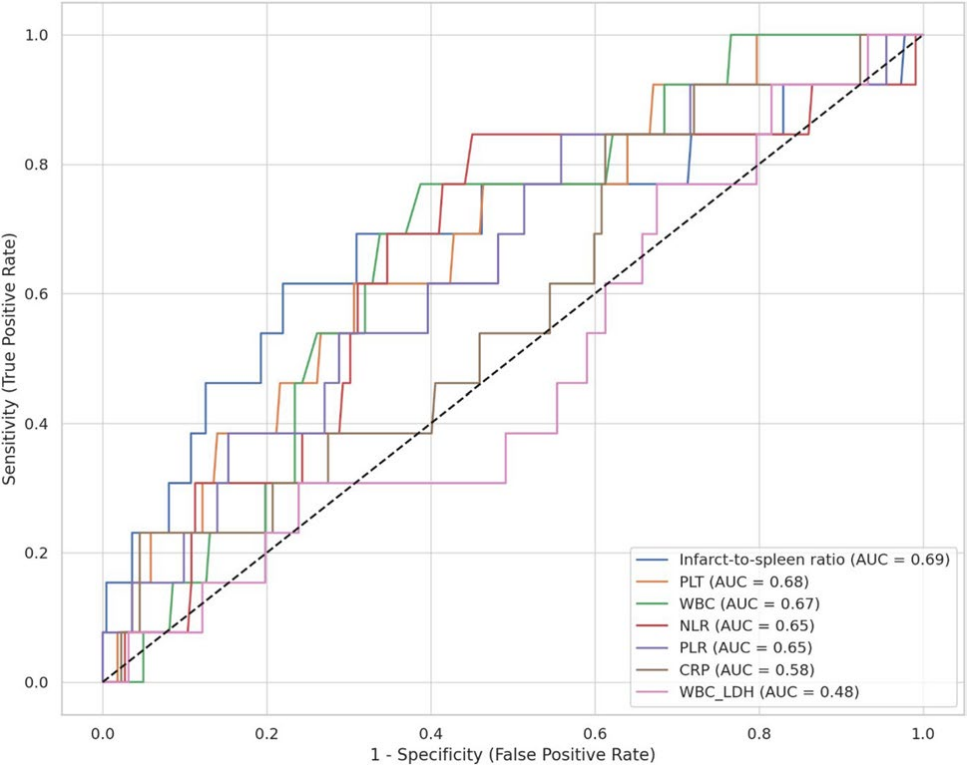
Receiver operating characteristic (ROC) curve of infarct-to-spleen volume ratio for predicting splenectomy in patients with splenic infarction

Receiver operating characteristic (ROC) curve analysis demonstrated that the ISR had the highest discriminatory performance for predicting surgical intervention, with an AUC of 0.69 (95% CI: 0.49–0.86), a specificity of 77.9%, and a negative predictive value (NPV) of 97.2%. Among hematological parameters, platelet count (PLT ≥ 300 × 10³/µL) yielded an AUC of 0.68 (95% CI: 0.53–0.82), with 69.4% specificity and 96.9% NPV. Other parameters showed lower AUC, sensitivity and specificity (Fig. 5) (Table 3). Univariate logistic regression analysis identified ISR (OR: 1.03, 95% CI: 1.01–1.05, *p* = 0.005) and fever (OR: 2.82, 95% CI: 1.46–5.44, *p* = 0.002) as significant predictors of surgical intervention. In the multivariate analysis, only ISR (OR: 1.03, 95% CI: 1.01–1.05, *p* = 0.002) and fever (OR: 2.69, 95% CI: 1.34–5.42, *p* = 0.005) remained independently associated with the likelihood of splenectomy (Table 4).

**Table 3:** Receiver operating characteristic (ROC) analysis of hematological and radiological parameters for predicting splenectomy in patients with Splenic infarction

Parameters	Cut-off value	AUC (95% CI)	Sensitivity %	Specificity %	PPV %	NPV %
ISR	25.9	0.69 (0.49–0.86)	61.5	77.9	14	97.2
PLT	300.0	0.68 (0.53–0.82)	61.5	69.4	10.5	96.9
WBC	10.8	0.67 (0.53–0.80)	76.9	61.3	10.4	97.8
NLR	5.0	0.65 (0.48–0.80)	84.6	55.0	9.9	98.4
PLR	145.8	0.65 (0.49–0.81)	84.6	44.1	8.1	98.0
CRP	20.0	0.58 (0.43–0.74)	84.6	38.7	7.5	97.7
WBC/LDH	0.01	0.48 (0.33–0.65)	92.3	18.5	6.2	97.6

*ISR* Infarct-to-spleen volume ratio, *PLT* Platelet count, *WBC* White Blood Cell count, *NLR* Neutrophil-to-Lymphocyte Ratio, *PLR* Platelet-to-Lymphocyte Ratio, *CRP* C-Reactive Protein, *WBC/LDH* White Blood Cell to Lactate Dehydrogenase Ratio

**Table 4:** Univariate and multivariate logistic regression analyses for identifying predictors of splenectomy in patients with Splenic infarction

Variable	Univariant			Multivariant		
	OR	95% CI	*P*	OR	95% CI	*P*
Age	0.98	0.01–1.79	0.124	-	-	-
ISR	1.03	1.01–1.05	**0.005**	1.03	1.01–1.05	**0.002**
Fever	2.82	1.46–5.44	**0.002**	2.69	1.34–5.42	**0.005**
WBC	1.04	0.97–1.11	0.252	-	-	-
NLR	0.98	0.90–1.08	0.732	-	-	-
PLR	1.00	1.00–1.08	0.101	-	-	-
CRP	1.00	0.99–1.01	0.674	-	-	-
WBC/LDH	0.00	0.00–4439	0.461	-	-	-

*ISR* Infarct/spleen volume Ratio, *PLT* Platelet count, *WBC* White Blood Cell count, *NLR* Neutrophil-to-Lymphocyte Ratio, *PLR* Platelet-to-Lymphocyte Ratio, *CRP* C-Reactive Protein, *WBC/LDH* White Blood Cell to Lactate Dehydrogenase Ratio

### Features of surgical group and follow-up

Table 5 reveals the characteristics of the patients who underwent splenectomy. All patients underwent splenectomy due to persistent abdominal pain that did not respond to narcotic analgesics. Atrial fibrillation was the most common (*n* = 5, 38.4%) etiological factor. Of those, two patients had previous thromboembolic events before splenic infarct. Two patients with advanced gastric cancer developed splenic infarct during oncologic treatment, and one patient with colon cancer developed during adjuvant chemotherapy. One 18-year-old female without comorbidities developed splenic infarction two days after undergoing cesarian section.

**Table 5:** Features of patients with splenectomy

Case#	Age	Gender	Clinical findings	Medical history	Etiology
1	58	M	LUQ pain Afebrile	Autoimmune hemolytic anemia	Hematologic disease
2	63	M	Diffuse abdominal pain Febrile	Gastric cancer	Non-hematological malignancy
3	18	M	LUQ pain Afebrile	None	Postoperative (cesarean section)
4	37	M	LUQ pain Afebrile	Granulomatosis with polyangiitis	Vasculitis
5	75	F	LUQ pain Subfebrile	HT, DM, Af	Atrial fibrillation
6	58	F	Diffuse abdominal pain Febrile	HT, Af, Mesenteric ischemia history	Atrial fibrillation
7	58	F	LUQ pain Afebrile	HT, CHF, Af	Atrial fibrillation
8	55	F	LUQ pain Febrile	HT, DM, CAD	Coronary artery disease
9	90	F	Diffuse abdominal pain Afebrile	Gastric cancer, HT, DM	Non-hematological malignancy
10	73	F	LUQ pain Subfebrile	HT, DM, pulmonary embolism history	Atrial fibrillation
11	77	M	LUQ pain Febrile	HT, DM, Colon cancer	Non-hematological malignancy
12	27	M	LUQ pain Febrile	SLE, HT	Autoimmune disease
13	30	M	Diffuse abdominal pain Subfebrile	HT, DM, CHF	Atrial fibrillation

*LUQ* Left upper quadrant, *HT* Hypertension, *DM* Diabetes mellitus, *Af* Atrial fibrillation, *CHF* Chronic heart failure, *CAD* Coronary artery disease, *SLE* Systemic lupus erythematosus

The median hospital follow up was also significantly longer in the surgical group (18 days [IQR: 15]) compared to the non-surgical group (2 days [IQR: 3.5], *p* = 0.004).

### Risk stratification model

**Fig. 6:**
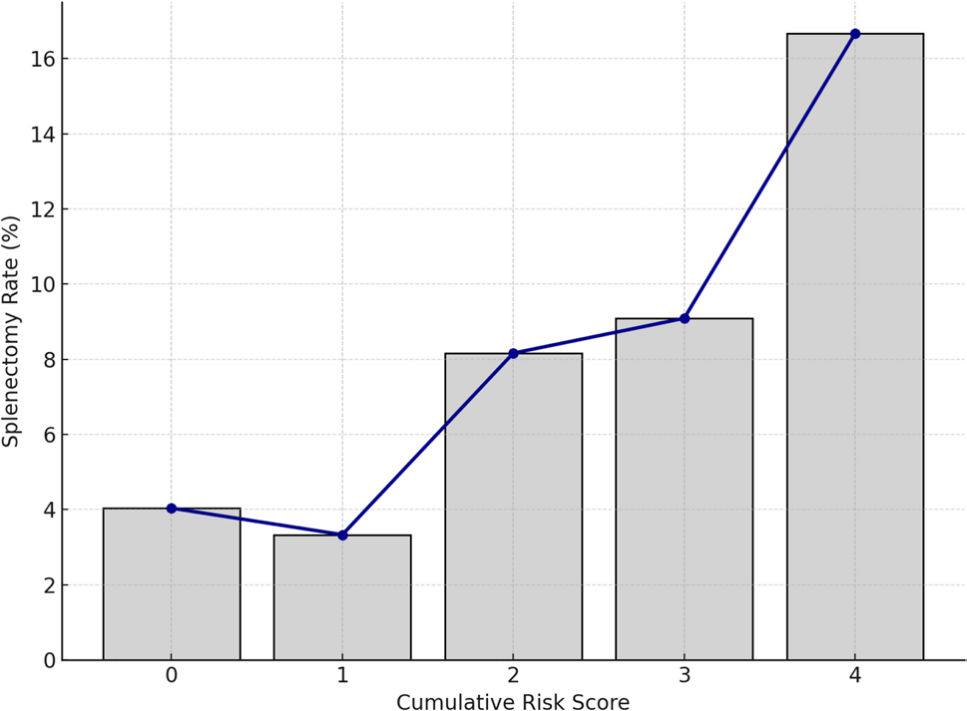
Distribution of splenectomies among cumulative risk groups

**Fig. 7:**
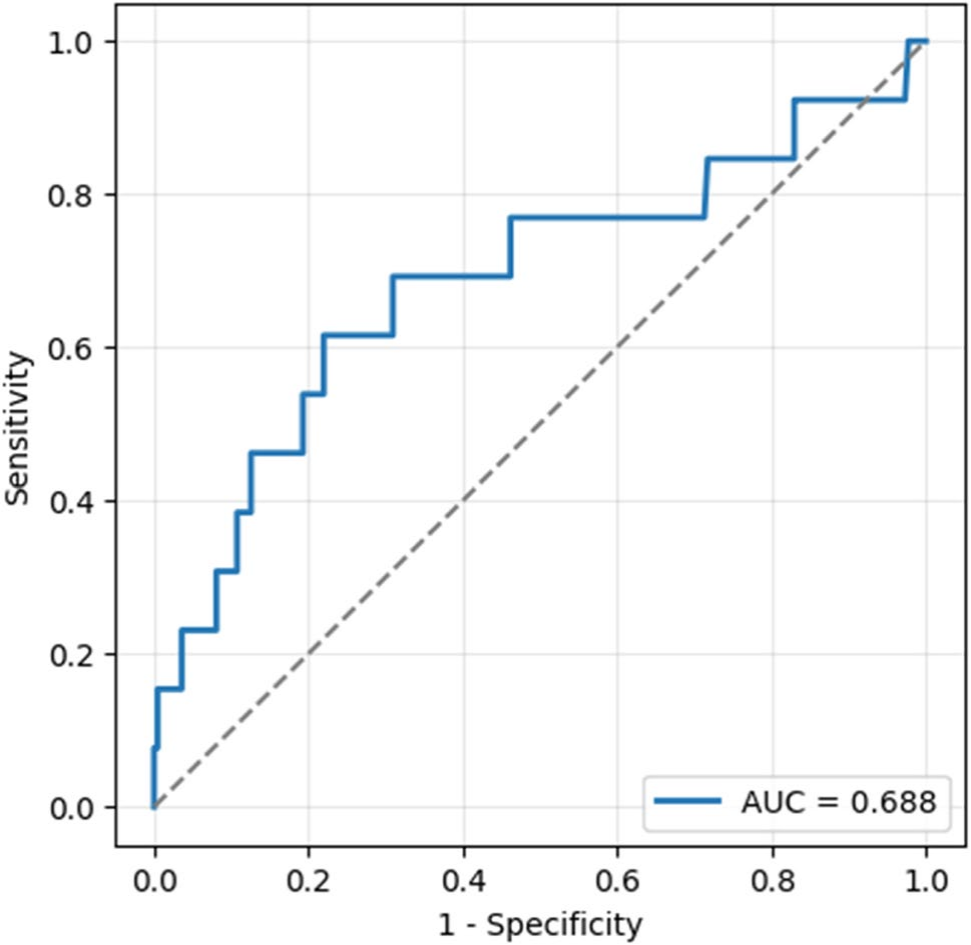
Receiver operating characteristic (ROC) curve of the risk prediction model for splenectomy

To develop a clinically practical prediction tool for splenectomy, we constructed a risk scoring system based on variables that were statistically significant in multivariate logistic regression analysis. Among all evaluated parameters, only the ISR and fever status were independently associated with the need for splenectomy. ISR was stratified into: <10 → 0 points, 10–25.9 → 1 point, > 25.9 → 2 points, based on the ROC-derived cut-off value. Fever was categorized as: Afebrile → 0 points Subfebrile → 1 point Febrile → 2 points, corresponding to the highest splenectomy incidence. A cumulative risk score ranging from 0 to 4 was calculated for each patient based on two independent predictors identified in multivariate analysis: ISR (0–2 points) and fever status (0–2 points). Patients were stratified into three risk categories: low risk (0–1 points), intermediate risk (2–3 points), and high risk (4 points) (Table 6). Of the 236 patients, 132 (55.9%) were classified as low risk, 95 (40.2%) as intermediate risk, and 9 (3.8%) as high risk. Splenectomy was performed in 2 patients (1.5%) in the low-risk group, 6 patients (6.3%) in the intermediate-risk group, and 3 patients (33.3%) in the high-risk group. A clear trend of increasing splenectomy rates with higher cumulative risk scores was observed (Fig. 6). The predictive model showed acceptable discrimination (AUC = 0.688). Calibration was excellent, as indicated by a non-significant Hosmer–Lemeshow test (χ² = 2.60, df = 8, *p* = 0.957). In addition, the Brier score was very low (0.049), supporting good overall accuracy of the model (Fig. 7).

**Table 6:** Risk groups based on ISR and fever score to guide follow-up and surgical need

Total Score	Risk group	Interpretation
**0–1**	Low risk	Outpatient follow-up may be appropriate
**2–3**	Intermediate risk	Close clinical monitoring is recommended
**4**	High risk	High likelihood of splenectomy; close observation needed

## Discussion

This retrospective cohort study revealed 5.6% splenectomy rate in patients with splenic infarcts who had high ISR. Multivariate logistic regression analyses also showed ISR more than 25.9% is an independent predictor for surgery. To the best of our knowledge, this is the first study in the literature to evaluate the ISR as a predictive marker for splenectomy in patients with splenic infarction. The ISR has been primarily studied in the context of partial splenic embolization, where it serves as a guide for achieving therapeutic efficacy while minimizing complications. Zhu et al. conducted a long-term study in 62 cirrhotic patients with hypersplenism undergoing partial splenic embolization and they reported that maintaining a splenic infarction rate between 50 and 70% yielded optimal hematological outcomes, including sustained improvements in platelet and leukocyte counts, while minimizing severe complications [11]. They also reported that patients with infarction rates above 70% had significantly higher rates of severe adverse events (50%) compared to those in the 50–70% range (approximately 9%). Similarly, Han et al., in a series of 30 patients treated with PSE alongside hepatic arterial embolization for hepatocellular carcinoma, demonstrated that infract more than 50% of splenic parenchyma was necessary to correct hypersplenism and they also found that patients with < 50% infarction did not achieve sustained hematologic improvement, whereas those with ≥ 50% infarction did—and without major complications [12]. Given the favorable clinical course, short inpatient stay, and lower extent of splenic involvement, our findings showed that splenectomy was performed in only 1.7% of the low-risk group compared to 13.8% and 33.3% in the intermediate and high-risk groups, respectively. With appropriate patient selection—particularly in those with small infarcts and stable vital signs—early discharge with structured outpatient follow-up may be a feasible and resource-efficient approach (Table 6).

In the retrospective series by Hakoshima et al., fever was observed in more than half of the patients with splenic infarction (57.1%) [4]. However, the authors emphasized that it remains unclear whether this febrile response was a direct manifestation of splenic infarction or secondary to the underlying etiologies such as infection, malignancy, or thromboembolic disease. This finding suggests that fever in splenic infarction should be interpreted cautiously, as it may reflect systemic inflammation driven by the primary condition rather than the infarction itself.

Splenic infarction remains a frequently missed diagnosis due to its vague and variable presentation; in up to one-third of cases, it is incidentally discovered on imaging without classical symptoms [4, 8, 13]. According to the existing literature, abdominal pain—particularly in the left upper quadrant —emerged as the most prevalent symptom in patients with splenic infarction and broad reviews of emergency department cohorts reported left-sided or LUQ pain in 20–65%, non-LUQ abdominal pain in 14–47%, and a subset (9–33%) were asymptomatic [9, 13, 14]. In our study, 69.2% of patients who underwent surgery had left upper quadrant and 30.8% had diffuse abdominal pain.

Although up to 20% of splenic infarctions may be asymptomatic and detected incidentally on CT imaging, they most commonly arise from emboligenic and thrombogenic conditions [2–4, 14]. In addition to atrial fibrillation, hematologic diseases—including myeloproliferative neoplasms and leukemias— and non-hematologic cancers contribute significantly, consistent with previous reports showing hematologic involvement in 10–59% of cases, and thrombosis risks ranging from 1% to 5% per patient-year in conditions such as essential thrombocythemia, primary myelofibrosis, acute leukemias, and lymphoma [1–3, 15–18]. Antineutrophil cytoplasmic antibody-associated vasculitis and antiphospholipid syndrome are also reported predisposing factors related to splenic infarction, as observed in our two patients secondary to granulomatosis with polyangiitis and systemic lupus erythematosus [19, 20].

Although splenectomy is infrequently required in cases of splenic infarction, surgical intervention is typically reserved for complicated or persistent presentations. Literature consistently suggests that most infarcts resolve with conservative, non-operative management, except in the presence of complications such as splenic rupture, abscess formation, hemorrhage, or a persistent pseudocyst [6, 7, 21]. Nores et al. and other series reported that septic emboli—often from endocarditis—and extensive infarction comprised a significant share of cases necessitating splenectomy, especially among hematologic malignancy patients, where complication rates may reach nearly 60% [6]. Additionally, persistent abdominal pain, fever, leukocytosis, and tachycardia have been identified as clinical indicators that are more common in patients requiring surgical management [21].

A key strength of this study is the incorporation of ISR as a quantitative radiologic parameter, which provides an objective and reproducible metric for assessing infarct burden and its association with surgical intervention—an aspect that has not been previously explored in the literature. On the other hand, our study had some limitations. First, this is a retrospective study based on medical records. Second, the retrospective nature of the study inherently limits data completeness, as some clinical variables were unavailable or inconsistently documented. Another limitation of our study is that we did not report outcomes of non-surgical patients according to risk categories due to lack of well documentation. Therefore, it remains unclear how different risk strata translate into clinical outcomes under conservative management, and our analyses should be regarded as exploratory.

In conclusion, this retrospective study suggests that the ISR may serve as a useful radiologic indicator associated with the likelihood of splenectomy in patients presenting with splenic infarction. Although many cases are managed conservatively, a greater infarct burden appears to correlate with increased surgical intervention rates. To our knowledge, this is the first study to explore ISR in this clinical context; however, these findings should be interpreted cautiously given the study’s design and sample characteristics. Selected patients with limited splenic involvement may be appropriate for outpatient follow-up, potentially avoiding unnecessary hospitalization. Future prospective studies with larger cohorts are warranted to confirm the predictive value of ISR and clarify its role in clinical decision-making.

## Data Availability

The data supporting the findings of this study are available from the corresponding author upon reasonable request. The dataset includes all raw data collected during the research, including participant responses and experimental results.

## Declarations

Each author hereby acknowledges that the final state of this manuscript is prepared and sent with his/her approval having been taken. The authors also confirm that this manuscript has not been published and is not under simultaneous consideration by another journal or electronic publication. All authors have read and complied with the requirements set forth in the Instructions to Authors.
